# Prioritizing Cancer Care in Low and Middle-Income Countries Using Delta Mortality-to-Incidence Ratios

**DOI:** 10.1200/GO.22.64000

**Published:** 2022-05-05

**Authors:** Thomas Diehl, Sheida Pourdashti, Daniel Schroeder, Syed Nabeel Zafar

**Affiliations:** ^1^ University of Wiscons, Madison, WI

## Abstract

Cancer outcomes are disparate around the world. Low- and middle-income countries (LMICs) face higher cancer mortality rates than high-income countries (HICs), and the burden will only intensify as cancer incidence is projected to rise 81% by 2040. Mortality-to-incidence ratios (MIR), calculated by dividing mortality rates by incidence rates, have been used to study disparities in cancer control. By calculating delta mortality-to-incidence ratios (dMIR), the difference between MIRs in HICs and LMICs, we can develop country-specific cancer priority lists for cancer control planning and resource allocation.

**METHODS:**

We extracted country-specific incidence and mortality rates for 35 cancer types from 183 countries using GLOBACAN 2020. Countries were grouped into income categories as defined by the World Bank. Development indicators and country metadata were extracted from the United Nations Development Program. MIRs were calculated for each cancer in every country. Linear regression was used to test relationships between MIRs and development indicators. Delta MIR was calculated for each cancer type by subtracting the average MIR for HICs from the average MIR for LMICs.

**RESULTS:**

For all cancers combined, MIRs varied widely across the globe, ranging from 0.33 in Australia to 0.80 in Gambia. Variation in MIR was low for certain diseases such as pancreas cancer (range = 0.80-1.0), but high for screenable cancers such as breast (0.11-0.63) and colon (0.28-1.0) cancers. Upon multivariate linear regression, MIRs were associated with life expectancy and income index. Cancers of the nasopharynx (0.90), Kaposi sarcoma (0.51), anus (0.46), salivary grands (0.38) and prostate (0.34) had the highest dMIRs.

**CONCLUSION:**

Delta MIRs can be employed to systematically address disparities in global cancer survival. Cancers with the highest dMIRs represent those with the greatest proportion of potentially avoidable deaths and should be targeted first for maximum impact on global cancer mortality. These data can inform country-specific cancer priority lists.

